# NeuO for Neuronal Labeling in Zebrafish

**DOI:** 10.18383/j.tom.2015.00127

**Published:** 2015-09

**Authors:** Chai Lean Teoh, Jun Cheng Er, Parag Mukherjee, Young-Tae Chang

**Affiliations:** 1 Singapore Bioimaging Consortium, Agency for Science, Technology and Research, Singapore;; 2 Graduate School for Integrative Sciences and Engineering, Centre for Life Sciences, National University of Singapore, Singapore; and; 3 Department of Chemistry and MedChem Program of Life Sciences Institute, National University of Singapore, Singapore

**Keywords:** BODIPY, fluorescent probes, imaging agents, live neurons, zebrafish

## Abstract

We report the application of our newly developed fluorescent probe 3-(benzylamino)-4,4-difluoro-5-(4-propyl-1*H*-1,2,3-triazol-1-yl)-8-(4-(2-hydroxyacetamido)phen-yl)-4-bora-3a,4a-diaza-*s*-indacene (NeuO) to label and image live neurons in zebrafish. Immersing zebrafish embryos in NeuO or injecting NeuO into zebrafish brain ventricles results in nontoxic in vivo neuronal labeling. We demonstrate the applicability of NeuO and envisage the potential of this compound as a rapid and simple labeling reagent for studying neuron development and degeneration.

## Introduction

Studying intact systems with molecular resolution and global scope remains a key challenge in biology. Among vertebrates, the zebrafish (*Danio rerio*) has become an attractive research model. They are not only inexpensive but also small, which makes them easy to maintain in large stocks. They also reproduce and grow quickly; a zebrafish can grow from a single cell to a swimming larva with a functional brain in less than 3 days. This compressed time scale allows scientists to quickly track its development. Zebrafish exhibit not only anatomic conservation in relation to mice and humans but also a high degree of genetic homology. In addition, because zebrafish are transparent during the first few days of their development, they can be imaged without further tissue manipulation.

The desired features of zebrafish combined with improvements in imaging techniques have facilitated zebrafish neuronal research. Multiple technical developments have permitted the visualization of zebrafish neuronal cell structures and their dynamics. In vitro approaches such as immunohistochemical analyses allow only the labeling of certain neuronal cell types (1), whereas ex vivo methods usually involve the application of membrane staining dyes such as DiI and DiO, which require manual injection for retrograde labeling (2). As an in vivo approach, stable transgenic zebrafish lines expressing fluorescent proteins that are driven by neuron-specific promoters have been employed. This method requires genetic manipulation, which in addition to being less straightforward can also be technically demanding (3).

Given this constraint, we sought to identify an alternative reagent to assess zebrafish neuronal structures. Small fluorescent molecules have been widely used as imaging probes to label proteins or specific cells. Recently, we communicated the discovery of a fluorescent compound, 3-(benzylamino)-4,4-difluoro-5-(4-propyl-1*H*-1,2,3-triazol-1-yl)-8-(4-(2-hydroxyacetamido)phen-yl)-4-bora-3a,4a-diaza-*s*-indacene (NeuO), to address existing limitations in live neuron imaging (4). NeuO was developed through a systematic screening of 5040 fluorescent compounds followed by a systematic structural optimization of the primary hit. We demonstrated that NeuO has an unprecedented ability to label and image live neurons selectively over other cells in the brain. It can be applied to multiple imaging and cellular platforms for the real-time imaging of neurons and is observed to be useful in vitro, ex vivo, and in vivo. The availability of NeuO with its superior versatility and ease of use therefore presents an attractive option to researchers in a myriad of neuronal targeting applications, such as in vivo imaging of zebrafish.

In this study, we explore the potential application of NeuO for in vivo neuronal labeling in zebrafish on the basis of our previous work. Of the 2 delivery strategies we investigated, one involved bathing zebrafish in a solution of NeuO; the other strategy involved directly injecting NeuO into the zebrafish brain ventricle space. We observed that, through immersion of zebrafish in NeuO, neurons located at the body surface, which include neurons of the nose, internal ear, and neuromasts, are all labeled intensely. In addition, NeuO injected into the brain ventricle labeled neuronal cells by diffusion and demonstrated that distinct cell shape changes—particularly in the brain and retina—occur during early zebrafish developmental stages.

## Materials and Methods

### Zebrafish Maintenance and Breeding

Wild-type AB-strain zebrafish were bred and maintained under standard conditions. Briefly, zebrafish were raised on a 14-h/10-h light/dark cycle at 28.5 ± 0.5°C. Embryos were obtained via natural mating and cultured in egg water. All experiments in this study were carried out according to the ethical guidelines established by the Institutional Animal Care and Use Committee. Embryos older than 24 hours postfertilization (pf) were treated with 200 μM 1-phenyl-2-thiourea to suppress pigment formation.

### Staining of Zebrafish by Immersion

Zebrafish larvae were immersed in egg water containing 10 μM NeuO for 30 minutes at room temperature. After a brief wash with egg water, the larvae were anesthetized with tricaine methanesulfonate (MS-222) and transferred onto glass-bottom petri dishes for imaging.

### Zebrafish Brain Ventricle Injection

The zebrafish brain ventricle injection method was conducted as previously reported (5). Briefly, chorions must first be removed from the embryos. The anesthetized embryos were mounted in 1% low-melting agarose in a tail-down orientation and further topped with some MS-222-containing egg water after the agarose had set. To inject the brain ventricle, the micromanipulation setup was arranged such that the needle tip was in the same field of view as the embryo. The needle was carefully put through the thin roof plate without hitting the brain tissue below. Just enough dye (10 μM NeuO, 1% dimethyl sulfoxide) was injected to completely fill the ventricles. Depending on the stage of the embryo, several injections may be needed. The embryos were observed under a fluorescence stereomicroscope for any incorrect injection and freed from agarose. Only the correctly injected embryos were selected to be remounted for confocal z-projection optical sectioning.

### In Vivo Imaging of Zebrafish

After being soaked with NeuO-containing medium or injected with NeuO, the zebrafish can be mounted and inverted in agarose to be imaged by confocal microscopy. To do so, a few drops of 1% low-melting agarose were laid over the zebrafish and immediately oriented as close to the bottom of the dish as possible. This was followed by droplets of MS-222-containing egg water over the set agarose surface. The embedded zebrafish were observed using a Nikon A1R^+^si confocal microscope with a 488-nm excitation laser and fluorescein isothiocyanate emission filter. Images were captured using a 4, 10, or 20× objective lens and processed with Nikon's NIS Elements software (Nikon Instruments, Melville, NY).

### Synthetic Procedures

#### 3-(Benzylamino)-4,4-difluoro-5-(4-propyl-1*H*-1,2,3-triazol-1-yl)-8-(4-nitrophenyl)-4-bora-3a,4a-diaza-*s*-indacene.

A solution of 3,5-dichloro-8-(4′-nitrophenyl)-4,4- difluoro-4-bora-3a,4a-diaza-s-indacence (15) (100 mg, 0.26 mmol) and NaN_3_ (0.17 g, 2.6 mmol) in a methanol (MeOH)/tetrahydrofuran (THF) (2:1) solvent mixture (5 mL) was stirred for 5 hours. Benzylamine (0.52 mmol, 60 μL) was added, and the reaction was further stirred for 1 hour. The crude mixture was concentrated in vacuo, redissolved in CH_2_Cl_2_, and quickly filtered through a short-path Al_2_O_3_ column. The filtrate was concentrated and redissolved in anhydrous THF under an argon environment. 1-Pentyne (0.78 mmol, 0.1 mL), Cu(ACN)_4_PF_6_ (10 mg, 0.026 mmol), and tris(benzyltriazolylmethyl)amine (15 mg, 0.026 mmol) were then added to the reaction mixture and stirred for 120 hours. Organic solvents were removed, and the resulting residue was diluted with water and extracted with CH_2_Cl_2_. The combined organic extracts were purified by flash column chromatography on silica gel to afford 3-(benzylamino)-4,4-difluoro-5-(4-propyl-1*H*-1,2,3-triazol-1-yl)-8-(4-nitrophenyl)-4-bora-3a,4a-diaza-*s*-indacene as an orange solid (23 mg, 0.044 mmol, 17% yield); ^1^H NMR (300 MHz, CD_3_CN) δ 8.33 (d, *J* = 8.8 Hz, 2H), 8.03 (s, 1H), 7.75 (d, *J* = 8.9 Hz, 2H), 7.54 (s, 1H), 7.45 – 7.25 (m, 6H), 7.00 (d, *J* = 5.2 Hz, 1H), 6.56 (d, *J* = 5.2 Hz, 1H), 6.49 (d, *J* = 3.8 Hz, 1H), 6.35 (d, *J* = 3.9 Hz, 1H), 4.67 (d, *J* = 6.3 Hz, 2H), 2.73 (t, *J* = 7.5 Hz, 2H), 1.73 (dq, *J* = 14.7, 7.4 Hz, 2H), 1.00 (t, *J* = 7.4 Hz, 3H); ^13^C NMR (126 MHz, CD_3_CN) δ 164.6, 149.5, 147.9, 141.3, 138.8, 137.6, 136.2, 135.8, 132.7, 131.6, 129.9, 128.8, 128.7, 128.0, 124.9, 124.6, 118.3, 116.3, 110.5, 48.6, 28.1, 23.5, 14.0, 1.3; HRMS (ESI+) calculated for C_27_H_24_BF_2_N_7_O_2_,[M+H]^+^: 528.2130, Found: 528.2136.

#### NeuO.

3-(benzylamino)-4,4-difluoro-5-(4-propyl-1*H*-1,2,3-triazol-1-yl)-8-(4-nitrophenyl)-4-bora-3a,4a-diaza-*s*-indacene (94 mg, 0.18 mmol) was dissolved in 10 mL of ethanol (EtOH) and 1 mL of acetic acid and heated to 90°C. A suspension of iron powder (280 mg, 5 mmol) was activated in 1 M HCl for 1 minute, rinsed with absolute EtOH, and added to the reaction mixture. After the reaction was completed the iron was removed, and the solvent was evaporated in vacuo. The residue was diluted with CH_2_Cl_2_ and washed with saturated NaHCO_3_. The organic extract was concentrated (∼10 mL of CH_2_Cl_2_) and treated with acetoxyacetyl chloride (90 μL, 0.9 mmol, 5 Eq) and 6 drops of saturated aqueous NaHCO_3_ and stirred at room temperature for 30 minutes. CH_2_Cl_2_ was subsequently removed in vacuo, and the reaction mixture redissolved in 10 mL of MeOH and 1 mL of water to which excess K_2_CO_3_ was added and stirred for 2 h. The reaction mixture was diluted with CH_2_Cl_2_, washed with water, and dried over anhydrous Na_2_SO_4_. The organic extract was evaporated and purified by flash column chromatography on silica gel to afford the title compound as an orange solid (61 mg, 0.11 mmol, 61% yield); ^1^H NMR (500 MHz, acetone-*d*_6_) δ 9.36 (s, 1H), 8.14 (s, 1H), 7.97 (s, 1H), 7.95 (d, *J* = 8.6 Hz, 2H), 7.52 (d, *J* = 8.6 Hz, 2H), 7.43 (d, *J* = 7.6 Hz, 2H), 7.37 (t, *J* = 7.6 Hz, 2H), 7.29 (t, *J* = 7.3 Hz, 1H), 7.11 (d, *J* = 5.1 Hz, 1H), 6.67 (d, *J* = 5.2 Hz, 1H), 6.51 (d, *J* = 3.8 Hz, 1H), 6.44 (d, *J* = 3.8 Hz, 1H), 4.96 (s, 1H), 4.87 (d, *J* = 6.5 Hz, 2H), 4.15 (s, 2H), 2.74 (t, *J* = 7.6 Hz, 2H), 1.75 (tq, *J* = 7.6, 7.4 Hz, 2H), 1.02 (t, *J* = 7.4 Hz, 3H); ^13^C NMR (126 MHz, acetone-*d*_6_) δ 171.5, 164.0, 147.4, 140.7, 139.3, 137.3, 136.7, 135.1, 132.2, 131.9, 131.3, 129.9, 129.7, 128.5, 127.9, 124.1, 120.1, 118.5, 114.9, 109.6, 63.1, 55.0, 28.3, 23.5, 14.2; HRMS (ESI+) calculated for C_29_H_28_BF_2_N_7_O_2_,[M+H]^+^: 556.2443, Found: 556.2461.

## Results and Discussion

**Scheme 1.**
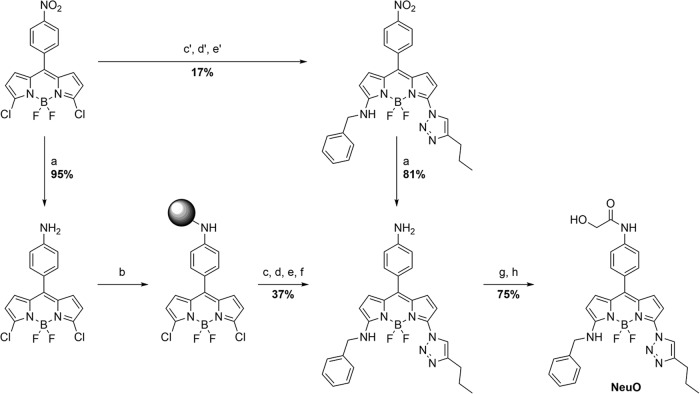
Synthesis of NeuO: conditions. Solid-phase method: (a) 1 M HCl-activated Fe, AcOH/EtOH (1:10), 5 minutes; (b) CTC-PS resin, DIEA, CH_2_Cl_2_/DMF, rt, 24 hours; (c) NaN_3_ (9 Eq), DMF, rt, 30 minutes; (d) benzylamine (10 Eq), DIEA/DMF (1:4), rt, 45 minutes; (e) 1-pentyne (10 Eq), CuI (4 Eq), l-AscA (4 Eq), rt, 30 minutes; (f) 0.5% TFA in CH_2_Cl_2_, 10 min; (g) (AcO)CH_2_COCl, saturated NaHCO_3_, CH_2_Cl_2_, 30 minutes; and (h) K_2_CO_3_, MeOH:H_2_O (10:1), 2 hours. Solution-phase alternative: (c′) NaN_3_ (10 Eq), MeOH:THF (2:1), rt, 5 h; (d′) benzylamine (2 Eq), 1 hour; and (e′) 1-pentyne (3 Eq), Cu(ACN)_4_PF_6_, TBTA, THF, rt, 120 hours. AcOH, acetic acid; CTC, chlorotrityl chloride; DIEA, diisopropylethylamine; DMF, dimethylformamide; PS, polystyrene; rt, room temperature; TFA, trifluoroacetic acid.

**Figure 1.**
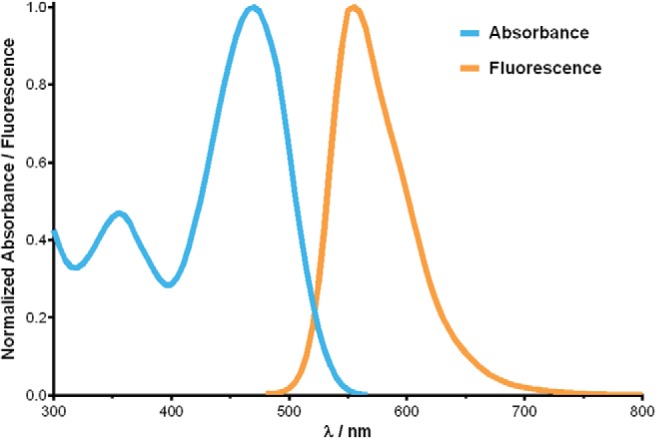
Optical properties of NeuO (10 μM in dimethyl sulfoxide; λ_ab, max_: 470 nm, λ_ex, max_: 555 nm).

Although NeuO is readily accessible using the solid-phase method, only limited quantities can be obtained; solid-phase yields decreased significantly when NeuO was synthesized at a scale larger than 10 mg. An alternative method using conventional solution-phase synthesis enables scale up, although overall percentage yields at larger scales are approximately 2.5 times lower than the solid-phase method (Scheme 1). Figure 1 illustrates the spectral properties of NeuO.

**Figure 2.**
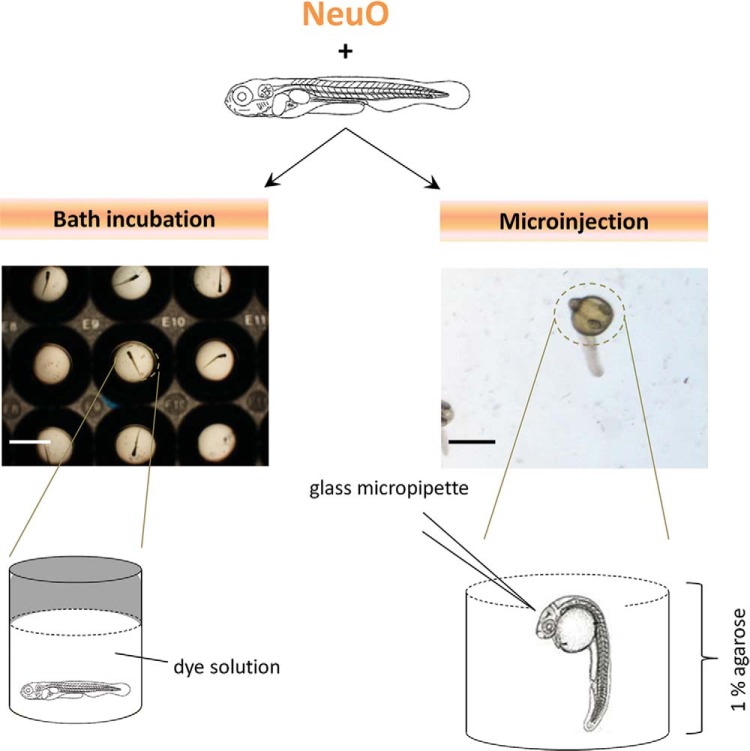
Delivery methods of NeuO to zebrafish. Using a 96-well plate, zebrafish embryos are immersed in dye solution within a well (left). Alternatively, zebrafish mounted in low-melting agarose contained within a petri dish were injected with a small quantity of dye solution into their brain ventricle (right). Scale bars = 200 μm.

To explore the capacity of NeuO in the imaging of zebrafish neuronal structures and to gain a fundamental understanding of the behavior of NeuO in vertebrate systems, we attempted 2 different delivery methods of the fluorescent dye, as illustrated in Figure 2. The first approach was more straightforward than the second and involved bathing zebrafish in a dye solution. Zebrafish larvae can absorb small molecules present in the surrounding water through their skin and gills. The second approach involved mounting zebrafish embryos in low-melting agarose contained within a petri dish to hold them in position, followed by the injection of a small quantity of dye solution into their brain ventricle space. The microinjection procedure is adapted and modified from a previously reported method (5).

**Figure 3.**
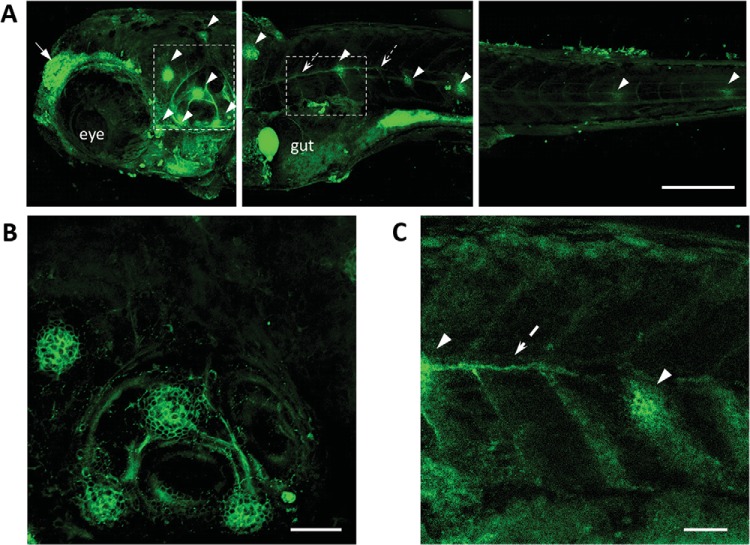
Zebrafish stained by NeuO after immersion in dye solution. (A) Lateral view. Single-focus fluorescence images from confocal stack of z-sections (total of 32), recorded in step sizes of 10 μm, are shown. Neurons of the nose (arrow), ear, and neuromasts of the lateral line (arrowheads) are clearly stained. The posterior lateral line nerve (dashed arrows), which innervates individual neuromast clusters, can also be observed by this method. (B, C) Magnified images of selected regions in (A). Single-focus fluorescence images from confocal stack of z-sections (total of 35), recorded in step sizes of 5 μm, are shown. Scale bars = 200 μm (A) and 50 μm (B, C), respectively.

Figure 3 shows 7-day pf zebrafish after immersion in 10 μM NeuO. Selective fluorescence staining of neurons by NeuO was observed and comparable to the localization of labeling presented in previous reports (4, 6). This includes the neurons of the nose, a paired organ located at the front end of the body, medial and anterior to the eyes, as well as neurons of the internal ear, which is located immediate caudal to the eye. The neuromasts are also labeled intensely by NeuO (Figure 3). Neuromasts, which are arranged on the body surface in a specific pattern, make up part of the lateral line system in zebrafish. The lateral line is a sensory system present in fish and amphibians that responds to changes in the motion of water and is involved in a variety of behaviors, from prey detection to predator avoidance, school swimming, and sexual courtship. As can be seen in the magnified images (Figure 3B; Supplemental Video 1), each neuromast in fact consists of a set of rosette-like organs, which is consistent with previous reports (7). In addition to labeling the individual neuromast cluster, labeling of the posterior lateral line nerve, which innervates these sensory organs, can also be observed (Figure 3C; Supplemental Video 2). Some traces of the dye detected in the gut area is likely to have resulted from the direct ingestion by the zebrafish during its incubation in the dye solution. Taken together, using this method, NeuO can label the neural cell bodies as well as the neuronal process and projections.

**Figure 4.**
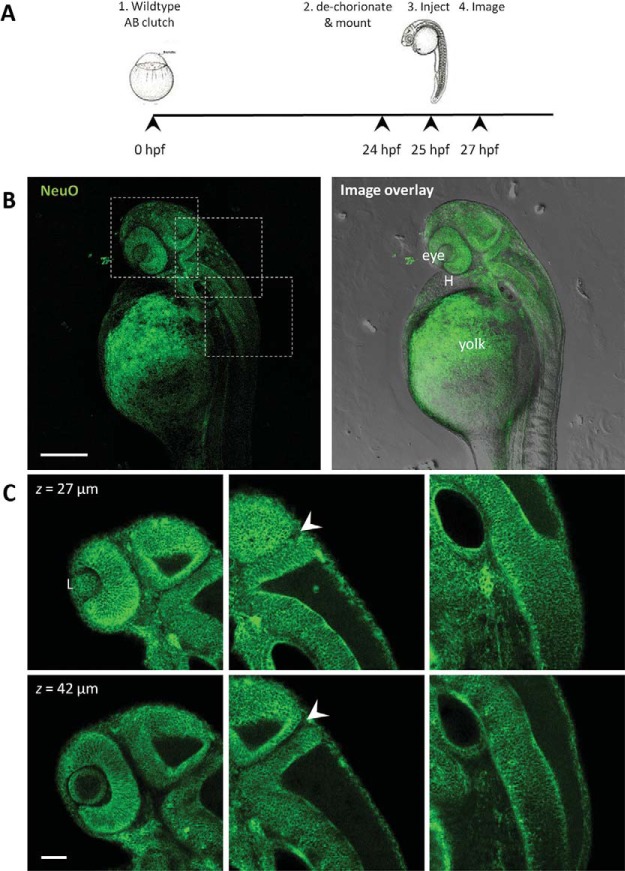
In vivo neuronal labeling by NeuO of 1-day postfertilization zebrafish. (A) Schematic of workflow. (B) Lateral view. A single-focus fluorescence image created from confocal stack of z-sections, recorded in step sizes of 10 μm, is shown (left) and overlaid with a differential interference contrast image (right). (C) Magnified images of selected regions in (B), showing 2 volume sections of a total of 48. A defined MHB can be observed (arrowheads). Scale bars are 200 μm (B) and 50 μm (C), respectively. Step size is 3 μm. H, heart; hpf, hours postfertilization; L, lens.

As a next step, we sought to deliver NeuO into the brain ventricle space of zebrafish embryos. The vertebrate brain, including that of fish and humans, originates from the neural tube. During vertebrate brain development, embryonic cerebrospinal fluid (eCSF) fills the center of the neural tube to form a system of cavities known as brain ventricles that is accompanied by the appearance of conserved folds and bends. eCSF and brain ventricles are essential for normal nervous system formation and function (8). The embryonic zebrafish brain is shaped as the ventricles within the neuroepithelium inflate. By 1 day pf, the initial steps of neural tube morphogenesis are complete (9). To prepare the embryo for microinjection at 1 day pf, chorions must first be removed from the embryos for the embryos to be mounted in low-melting agarose in a tail-down orientation. After injection, the zebrafish are checked under a stereomicroscope, where only the correctly injected embryos are selected to be remounted for confocal z-projection optical sectioning (Supplemental Figure 1) (5). A schematic of the workflow is shown in Figure 4A.

Figure 4B shows the distribution of NeuO in the 1-day pf zebrafish embryo after delivery. The compound was found to concentrate in the anterior region of the zebrafish. Fluorescence signaling detected in the yolk was considered to be an intrinsic contribution that can also be observed in tissues without NeuO under the same imaging parameters (data not shown). Selected confocal z-projection volume sections from an enlarged area of the zebrafish eye shows that the lens is spherical and has detached from the epidermis (Figure 4C; Supplemental Video 3). Early development of the zebrafish eye has been described in detail previously (10). The vertebrate retina develops from a single sheet of neuroepithelial cells that later differentiate and reorganize into layered structures during retinal neurogenesis. At this stage, we can see that there are not yet any clearly distinguishable neuronal cell layers.

Using this method, it is apparent that the injected NeuO liquid has successfully dispersed throughout the eCSF and taken up by cells close to the ventricle, where individual cells of the neuroepithelium have been distinctly labeled by NeuO. The brain cells are observed to be narrow, long, and spindly, which is similar to the observations reported in previous studies in which transgenic zebrafish lines that express fluorescent proteins were used (Figure 4C; Supplemental Videos 4 and 5) (9, 11). Another observable feature is the constriction of the neuroepithelium in which regions of the neuroepithelium bend sharply and form the prominent fold referred to as the midbrain-hindbrain boundary (MHB). The MHB is the site of one of the earliest bends in the developing brain. These folds and bends delineate the functional unit of the brain (12), where the process involves cell shape changes and cytoskeletal rearrangements. Such organization has been reported to be an integral part of a functional organ (11).

**Figure 5.**
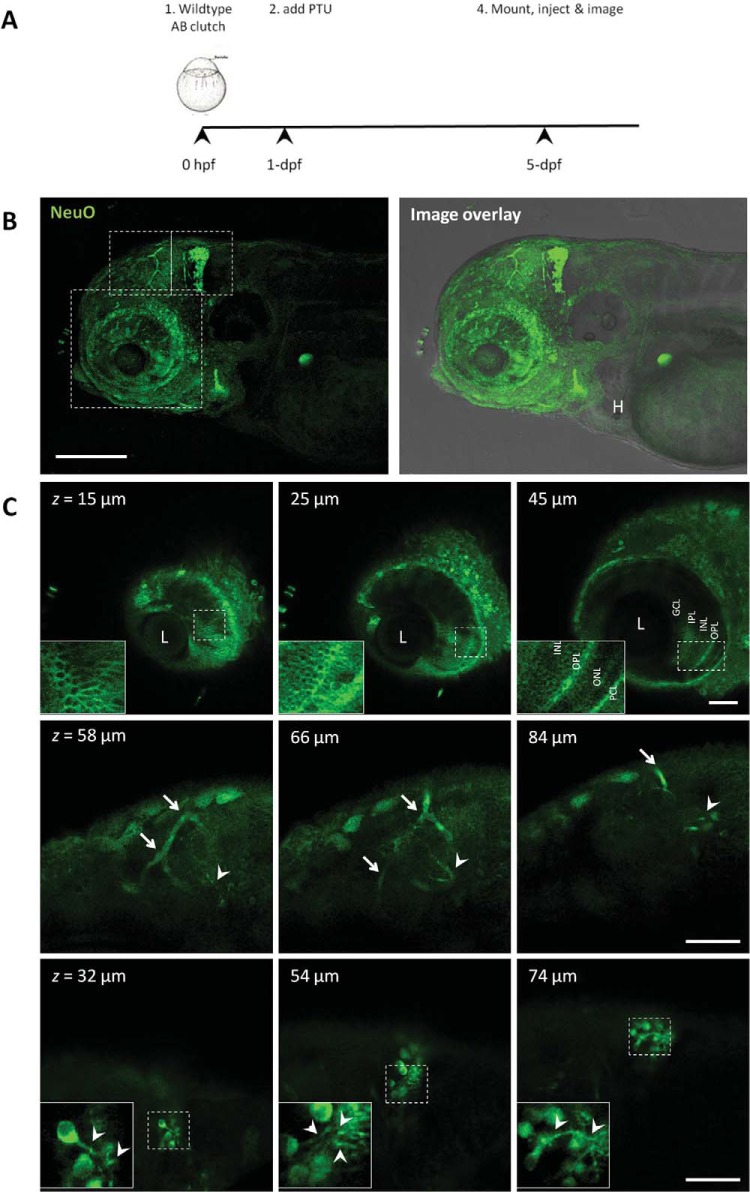
In vivo neuronal labeling by NeuO of 5-day postfertilization zebrafish. (A) Schematic of workflow. (B) Lateral view. A single-focus fluorescence image created from confocal stack of z-sections, recorded in step sizes of 10 μm, is shown (left) and overlaid with a differential interference contrast image (right). (C) Magnified images of selected regions in (B), showing 3 volume sections of a total of 37, 67, and 59, respectively. Step size is 5 μm (top) and 2 μm (middle, bottom). Areas of interest are shown as insets. Blood vessels (arrows) and neuronal projections (arrowheads) are as indicated. Scale bars = 200 μm (B) and 50 μm (C), respectively. dpf, days postfertilization; H, heart; hpf, hours postfertilization; L, lens.

Encouraged by this result, we repeated the microinjection process on more developed 5- day pf zebrafish (Figure 5A). At 5 days pf, the larval brain is less than 500 μm thick and 1.5 mm long (13). By this stage, the forebrain ventricle space is no longer visible under the stereomicroscope as described previously (1 day pf). As such, we mounted the zebrafish similarly but directed the injection site toward the midline of the midbrain. Figure 5B shows the distribution of NeuO in the 5-day pf zebrafish 2 hours after injection. Intense NeuO labeling can be observed around the site of injection, and some diffusion of the dye toward the forebrain, eyes, and jaw area can also be observed. Selected confocal z-projection volume sections from each area are shown in Figure 5C.

Unlike the earlier observation that neuronal cell layers are not yet clearly distinguishable in the retina of 1-day pf zebrafish, we can now visualize the development of layered structures that are labeled by NeuO (Figure 5C; Supplemental Video 6). Retinal neurons are composed of specific neuronal classes that execute distinct functions. These classes are organized into 3 major nuclear layers: the ganglion cell layer, inner nuclear layer, and outer nuclear layer. These 3 laminae are separated by 2 plexiform layers—the inner and outer plexiform layers—that mainly contain neuronal projections (14). Although the heart and most circulation remain unstained, NeuO signaling can be observed in certain areas of the brain vasculature (Figure 5C; Supplemental Video 7). Similar to our previous report (4), a distinct cytoplasmic perinuclear staining pattern of neuronal cell bodies by NeuO can be seen around the site of injection upon close observation (Figure 5C; Supplemental Video 8). In addition, NeuO staining also extends to the fine processes of the neurites. It is unclear why only a limited subset of neurons nearest to the injection site has been labeled preferentially here, whereas NeuO labeling of the neuroepithelium in 1-day pf zebrafish is well distributed. One possible explanation may be the better distribution of the dye through eCSF when injected into the ventricle space, which was impeded in 5-day pf zebrafish larvae. Labeling by the immersion method is limited to the surface neurons only; otherwise, NeuO will stain neurons without preference for a particular neuronal subtype (4). Although NeuO seems to be stable in vivo, the compound is limited in its application for prolonged imaging. Because the dye signal is no longer detectable >20 hours after injection or incubation (data not shown), imaging by NeuO is best attempted within 2 to 5 hours after delivery. NeuO is not fixable and is thus limited to live cells only (4).

In summary, we demonstrated that neuronal labeling can be obtained by simple short-term soaking of embryos in NeuO-containing medium or by delivery into the brain ventricle space via injection. The availability of NeuO with its superior versatility and ease of use, combined with more strategic designs underway in our group, will therefore set the basis for developing NeuO as a valuable tool for future neuronal targeting applications. Additional efforts to elucidate the binding mechanism of NeuO are in progress.

### Supplemental Materials

Video 1:

Video 2:

Video 3:

Video 4:

Video 5:

Video 6:

Video 7:

Video 8:

Supplemental Figure:
