# Supplementary material for: NeuO for Neuronal Labeling in Zebrafish
Source: Tomography. 2015 Sep;1(1):30–6. doi: 10.18383/j.tom.2015.00127 (PMC6024405; doi:10.18383/j.tom.2015.00127)
Supplement: Supplemental Figure: [file tom-01-01-s009.pdf]

NeuO for Neuronal Labelling in Zebrafish.

Chai Lean Teoh[1], Jun Cheng Er[2], Parag Mukherjee[3] and Young-Tae Chang*[1,3]

[1] Singapore Bioimaging Consortium, Agency for Science, Technology and Research (A*STAR), 138667, Singapore;

[2] Graduate School for Integrative Sciences and Engineering, National University of Singapore, Centre for Life Sciences, #05-01, 28 Medical Drive, 117456, Singapore;

[3] Department of Chemistry & MedChem Program of Life Sciences Institute, National University of Singapore, 117543, Singapore;

Supplementary Information

Figure S2. Microinjection efficiency of **NeuO** into zebrafish (1-dpf) brain ventricle.

Supplementary videos legends.

Synthetic procedures

Supplementary Figures

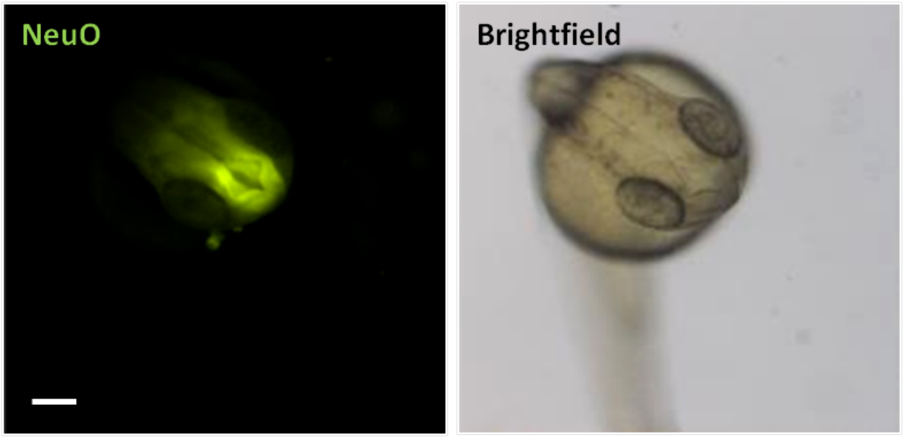

Figure S1. Microinjection efficiency of **NeuO** into zebrafish (1-dpf) brain ventricle. Stereomicroscope fluorescence image (*left*) and brightfield image (*right*) of zebrafish with **NeuO** injected into its brain ventricle space. Scale bar represents 200 µm.

Supplementary Videos Legends

Zebrafish stained by NeuO after immersion in dye solution.

Video S1. Confocal stack of z-sections (total 35) in Figure 2B merged with DIC images, recorded in step size of 5 μm. Rosette-like appearance of each neuromast can be clearly seen.

Video S2. Confocal stack of z-sections (total 35) in Figure 2C merged with DIC images, recorded in step size of 5 μm. Posterior lateral line (pLL) nerve (dashed arrows), which innervates individual neuromasts clusters can be observed.

Zebrafish (1 dpf) *in vivo* neuronal labelling by NeuO.

Video S3. Confocal stack of z-sections (total of 48) in Figure 3C (*left*) merged with DIC images, recorded in step size of 3 μm. Enlarged area of the zebrafish eye shows that the lens is spherical and has detached from the epidermis. None of the retina neuronal cell layers are clearly distinguishable yet.

Video S4. Confocal stack of z-sections (total of 48) in Figure 3C (*middle*) merged with DIC images, recorded in step size of 3 μm. Defined mid-brain and hind-brain boundary (MHB) can be observed.

Video S5. Confocal stack of z-sections (total of 48) in Figure 3C (*right*) merged with DIC images, recorded in step size of 3 μm.

Zebrafish (5 dpf) *in vivo* neuronal labelling by NeuO.

Video S6. Confocal stack of z-sections (total of 37) in Figure 4C (*top*) merged with DIC images, recorded in step size of 5 μm. Layered structures in the retina of zebrafish that are labeled by **NeuO** can be visualized.

Video S7. Confocal stack of z-sections (total of 67) in Figure 4C (*middle*) merged with DIC images, recorded in step size of 2 μm. **NeuO** signal can be observed in certain area of the brain vasculature.

Video S8. Confocal stack of z-sections (total of 59) in Figure 4C (*bottom*) merged with DIC images, recorded in step size of 2 μm. A distinct cytoplasmic perinuclear staining pattern of neuronal cell bodies by **NeuO** can be observed. **NeuO** staining also extends to the fine processes of the neurites.
